# Community differences and potential function along the particle size spectrum of microbes in the twilight zone

**DOI:** 10.1186/s40168-025-02116-8

**Published:** 2025-05-14

**Authors:** Yue Zhang, Hongbin Liu, Hongmei Jing

**Affiliations:** 1 Institute of Deep-Sea Science and Engineering, Chinese Academy of Sciences, Sanya, China; 2 Department of Ocean Science, The Hong Kong University of Science and Technology, Clear Water Bay, Kowloon, Hong Kong SAR, China; 3 HKUST-CAS Sanya Joint Laboratory of Marine Science Research, Chinese Academy of Sciences, Sanya, China; 4 Southern Marine Science and Engineering Guangdong Laboratory, Zhuhai, 519000 China

**Keywords:** Twilight zone, Prokaryotes, Community differences, Ecological function

## Abstract

**Background:**

The twilight zone, which extends from the base of the euphotic zone to a depth of 1000 m, is the major area of particulate organic carbon (POC) remineralization in the ocean. However, little is known about the microbial community and metabolic activity that are directly associated with POC remineralization in this consistently underexplored realm. Here, we utilized a large-volume *in situ* water transfer system to collect the microbes on different-sized particles from the twilight zone in three regions and analyzed their composition and metabolic function by metagenomic analysis.

**Results:**

Distinct prokaryotic communities with significantly lower diversity and less endemic species were detected on particles in the South East Asian Time-series Study (SEATS) compared with the other two regions, perhaps due to the *in situ* physicochemical conditions and low labile nutrient availability in this region. Observable transitions in community composition and function at the upper and lower boundaries of the twilight zone suggest that microbes respond differently to (and potentially drive the transformation of) POC through this zone. Substantial variations among different particle sizes were observed, with smaller particles typically exhibiting lower diversity but harboring a greater abundance of carbon degradation-associated genes than the larger particles. Such a pattern might arise due to the relatively larger surface area of the smaller particles relative to their volume, which likely provides more sites for microbial colonization, increasing their chance of being remineralized. This makes them less likely to be transferred to the deep ocean, and thus, they contribute more to carbon recycling than to long-term sequestration. Both contig-based and metagenome-assembled genome-(MAG-) based analyses revealed a high diversity of the Carbohydrate-Active enZymes (CAZy) family. This indicates the versatile carbohydrate metabolisms of the microbial communities associated with sinking particles that modulate the remineralization and export of POC in the twilight zone.

**Conclusion:**

Our study reveals significant shifts in microbial community composition and function in the twilight zone, with clear differences among the three particle sizes. Microbes with diverse metabolic potential exhibited different responses to the POC entering the twilight zone and also collectively drove the transformation of POC through this zone. These findings provided insights into the diversity of prokaryotes in sinking particles and their roles in POC remineralization and export in marine ecosystems.

Video Abstract

**Supplementary Information:**

The online version contains supplementary material available at 10.1186/s40168-025-02116-8.

## Introduction

Organic matter produced in the surface ocean is exported to the interior by the biological carbon pump in both particulate and dissolved forms [1, 2]. POC is composed of zooplankton fecal pellets [3] and organic aggregates of various sources [4, 5]. These carbon-rich particles provide a substrate for microbial colonization and degradation on their way to the deep ocean. This is an important process in the remineralization and cycling of nutrients. The particles that are not utilized fall to the seafloor where they are buried and stored for a long period of time, and so these are an important sink of anthropogenic CO_2_.

The oceanic “twilight” zone is usually defined as the layer below the epipelagic zone (i.e., it extends from a depth of ca. 100–200 m to ca. 1000 m); it is an oxygen minimum zone, with low dissolved organic matter (DOM) and a high concentration of inorganic nutrients [6]. This realm acts as an important conduit for POC that sinks from the surface of the ocean into deeper waters, and it determines the efficiency of the biological carbon pump [7]. More than 70% of the POC that sinks is remineralized in the twilight zone. This is due to biological processes, such as fragmentation and repackaging by zooplankton and decomposition and remineralization by microbial metabolism, as it sinks through the water column [8]. In addition, the high demand for carbon in the twilight zone is sustained by various other carbon supply pathways, such as lateral transport, dark carbon fixation, and the active transport of carbon due to the migration of zooplankton [9]. Consequently, the twilight zone determines the amount of biological carbon that eventually reaches the bottom of the ocean where it is stored; hence, it helps to regulate the global climate.

The fraction of exported particles that reach the interior of the ocean is also controlled by biotic (mainly microbiome and zooplankton) transformations during their downward transit, and this also affects their remineralization rate and the attenuation of POC flux with depth [10, 11]. In the past two decades, the number of studies that describe the role of microbes in the biological remineralization of POC in the twilight zone has increased [12, 13]. These studies report the growing evidence that heterotrophic prokaryotes are responsible for the decomposition and remineralization of approximately 70–92% POC in the twilight zone, and thus, they influence the efficiency of both the biological and microbial carbon pumps [14, 15]. Variations in the net primary production and phytoplankton community structure in the ocean likely affect the efficiency of POC sinking export, resulting in differences in the composition and function of microbial communities in the twilight zone [16]. For example, studies on the mesopelagic zone revealed that microbial groups with specific metabolic capabilities might be involved in POC remineralization [17]. However, information regarding the taxonomic composition and metabolic activity of prokaryotic communities involved in the remineralization of POC in the twilight zone is still lacking [13], and whether there is a shift between the upper and lower boundaries of this realm is largely unknown.

Particle association is reported to be a significant factor in microbial distribution, community composition, and activity [18]. For example, prokaryotes have traditionally been classified into free-living (FL) or particle-attached (PA) forms. A wide range of filters are available to separate the various FL and PA fractions, including 0.8 μm [19], 1.6 μm [20], 3.0 μm [21], 5.0 μm [22], and even 30 μm [23] pore sizes. Using this separation method, the bacterial diversity and community structure of six discrete size fractions, from 0.2 to 200 μm, were shown to be distinct [24]. This reflects the complexity of size selection on the evaluation of the diversity and function of PA microbes in ecological studies. On the other hand, trap-collected particles are often classified into the following size fractions: 1–10 µm, 10–50 µm, 50–150 µm, and > 150 μm, for biogeochemical studies on the composition and flux of different-sized sinking particles [25–27]. In general, small-sized (i.e., < 50 μm) sinking particles, especially those of 1–10 μm, have been reported to dominate the bulk ^234^Th and POC flux in the northern South China Sea (SCS) [27]. Moreover, particles < 64 μm were described as being an important contributor to the flux of carbon throughout the mesopelagic zone in the Sargasso Sea [28]. Therefore, to better understand the role of different size-fractionated PA microbes in the twilight zone, it is necessary to use a biogeochemical classification method instead of the traditional biological size method to track the population migration and potential functions of microorganisms in different size trap-collected particles.

In the present study, a large volume *in situ* water transfer system was applied to enrich and identify microbes associated with particles > 50 µm, 10–50 µm, and 1–10 µm at the upper and lower boundaries of the twilight zone in three distinct regions (i.e., the SEATS, two cold seeps, and one hydrothermal vent), and they were compared with those from the euphotic layer. A metagenomic study and bioinformatic analysis were used to reveal the composition and metabolic function of the microbial communities associated with the particles, and MAGs were obtained to reconstruct the metabolic potential of the microbiome. The objective of this study was to better understand the shifts and connectivity of the microbial communities in the twilight zone, especially with regard to their metabolic capabilities in terms of degradation, aggregation, and transformation of organic matter.

## Results

### Hydrographic conditions

**Fig. 1:**
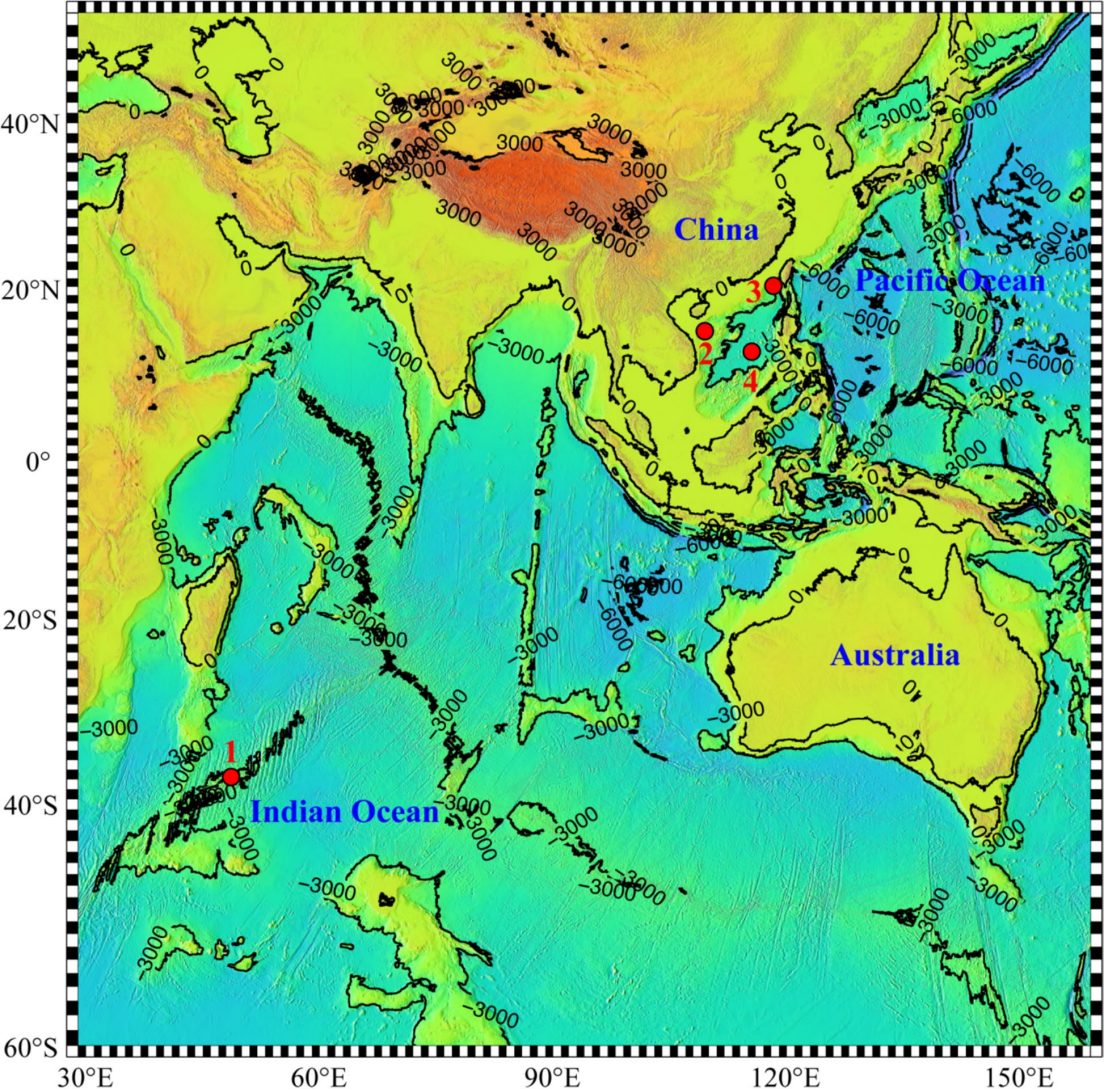
Map of the sampling habitats used in this study. “1” represents Longqi station, located in the Southwest Indian Ocean (SWIO); “2” and “3” represent Haima and Site F stations, respectively, in the northern South China Sea (NSCS); and “4” represents the South East Asian Time-series Study (SEATS) station in the South China Sea

The NSCS (i.e., Haima and Site F, northern South China Sea) exhibited higher nitrate and POC concentrations than the SEATS and SWIO (Longqi, Southwest Indian Ocean) in the twilight zone (Fig. S1; Table S1). In addition, station Haima of the NSCS had the lowest water temperature. The SWIO exhibited a higher temperature at 800 m, as well as markedly lower concentration of silicate (0.70–1.16 µmol/L) than that of the other regions. In the NSCS, higher levels of ammonia and DOC were detected at the upper boundary of the twilight zone than at the lower boundary, whereas an opposite pattern was observed regarding the concentrations of phosphate and silicate. In the SEATS and SWIO sites, the amount of POC decreased with depth, whereas the levels of phosphate and nitrate were both highest at the lower boundary of the twilight zone (Fig. 1).

### Population shifts in the microbes associated with particulate organic matter (POM)

**Fig. 2:**
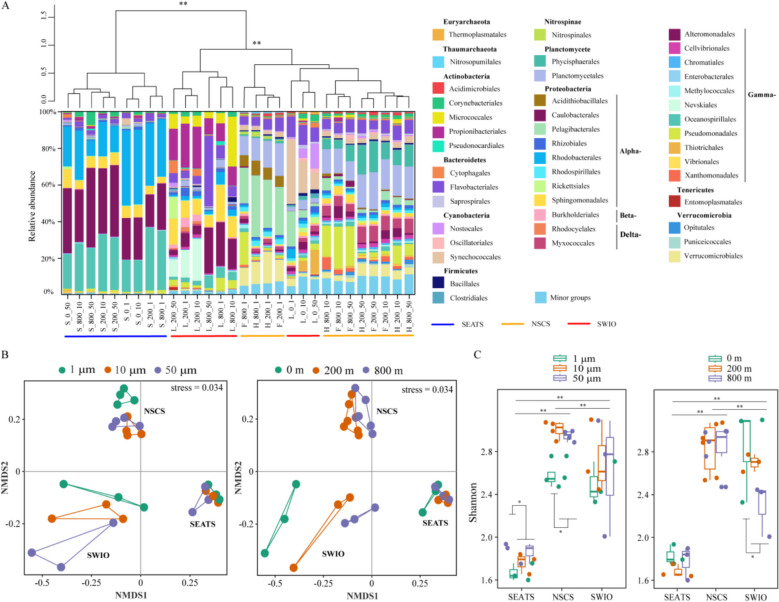
Composition and diversity of prokaryotic communities among the different sampling regions. **A** Taxonomic composition at the order level with a minimum of 1% of the total sequences. In the sample names, the uppercase letters denote the station names, such that S, H, F, and L indicate SEATS, Haima, Site F, and Longqi, respectively. The second number indicates the sampling depth, and the third number represents the particle size fraction. **B** nMDS ordination based on the Bray–Curtis dissimilarity, showing the clustering of different size fractions (left) and water depth (right) at the order level. The samples are color-coded accordingly. Significant clusters were determined by ANOSIM (9999 permutations, *p* < 0.01). NSCS indicates Haima and Site F sampling stations, whereas SWIO indicates Longqi sampling site. **C** The Shannon index of the prokaryotic community according to the fraction size (left) and water depth (right) in the different sampling regions

After trimming and assembly, a total of 12,351,544 contigs were obtained, ranging from 199,531 to 736,345 contigs in each sample, with an average GC% content of 49.7% (Table S2). Proteobacteria, particularly the Alphaproteobacteria and Grammaproteobacteria, were the predominant groups throughout the study areas; they occupied higher proportions in the 1- to 10-µm fraction and at a depth of 800 m than in the other particle fractions and depths in each region (Fig. 2A). Based on our unweighted pair group method with arithmetic mean (UPGMA) (Fig. 2A) and nonlinear multidimensional scaling (nMDS) (Fig. 2B) analyses, the structure of the prokaryotic community in the water column of the SEATS was found to be distinct from that of the other two regions.

A clear differentiation of prokaryotic microbial taxa was observed between the smallest particles (i.e., 1–10 µm) and the larger particles (i.e., 10–50 μm and > 50 μm) in both NSCS and SEATS. For example, Pelagibacterales appeared to be associated with the small particles, whereas Alteromonadales were found more frequently in the larger particles. In contrast, a depth-dependent distribution of prokaryotic taxa was evident in the SWIO. For example, Thiotrichales were most abundant in the surface water, Propionibacteriales and Cytophagales were more commonly found at the upper boundary, and Rhodobacterales, Alteromonadales, Oceanospirillales, and Opitutales were more prevalent at the lower boundary of the twilight zone (Fig. 2A). Regarding community diversity of the different-sized particles among the three regions, the lowest occurred in the 1- to 10-µm particle fraction of the SEATS. In addition, when comparing the 1–10 µm and larger fractions, the diversity was significantly different in the NSCS and SEATS (Fig. 2C, left graph). Moreover, the highest diversity was found in the surface water of the SWIO, and it was distinct from those at the upper and lower boundaries of the twilight zone (Fig. 2C, right graph).

**Fig. 3:**
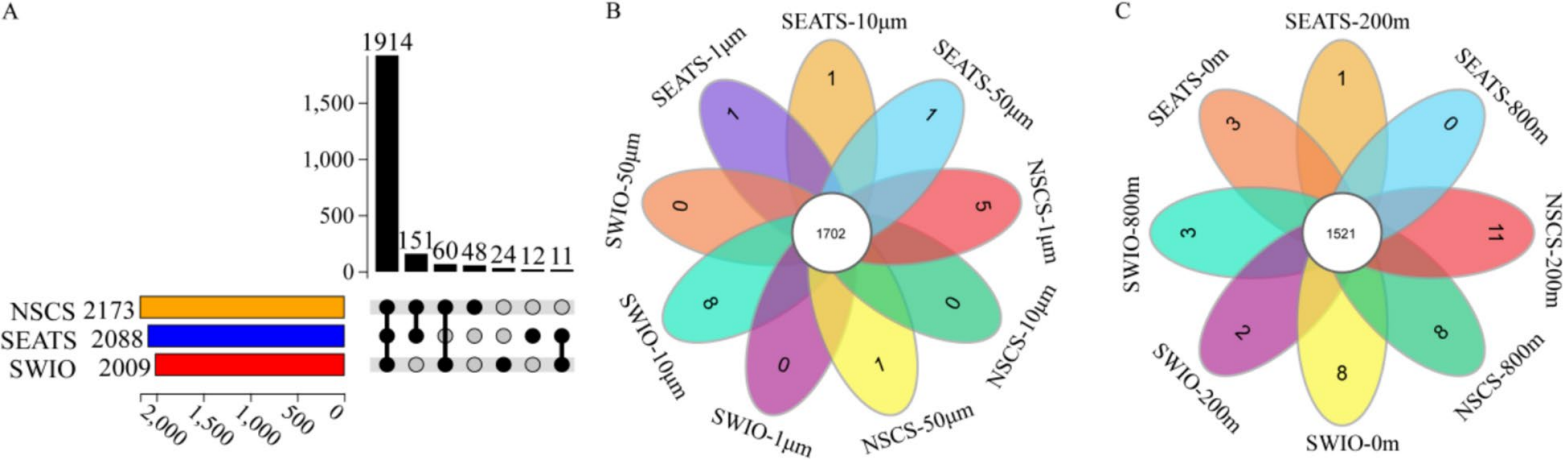
**A** An UpSet plot illustrating the distribution of taxonomic groups across the three sampling regions. The black circles and vertical lines represent the intersections between taxonomic groups corresponding to each vertical bar. Petal diagrams showing the number of genera shared among **B** the different size fractions and **C** the water depths. NSCS indicates Haima and Site F, whereas SWIO indicates Longqi

The UpSet diagrams demonstrate that 1914 taxonomic sequences at the genus level were shared among the 3 regions (Fig. 3A). When investigating particle size and depth, the highest numbers of specific genera occurred in the 10- to 50-μm particles in the SWIO (Fig. 3B) and at the upper boundary of the twilight zone in the NSCS (Fig. 3C), respectively. The prokaryotic taxa that were predominant in each of the three regions were identified by LEfSe. We found that Planctomycetales, Verrucomicrobiales, and Myxococcales were enriched in the NSCS, whereas Alteromonadales, Oceanospirillales, and Rhodobacterales were the most important taxa in the SEATS, and Propionibacteriales and Micrococcales were more populous in the SWIO (Fig. S2A). Moreover, our STAMP analysis showed that in both NSCS and SWIO, the indicative groups that resulted in significant differences between the upper and lower boundaries of the twilight zone were Thermoanaerobacterales and Deferribacterales, respectively (Fig. S2B and C).

### Interaction among microbes and environmental impact

**Fig. 4:**
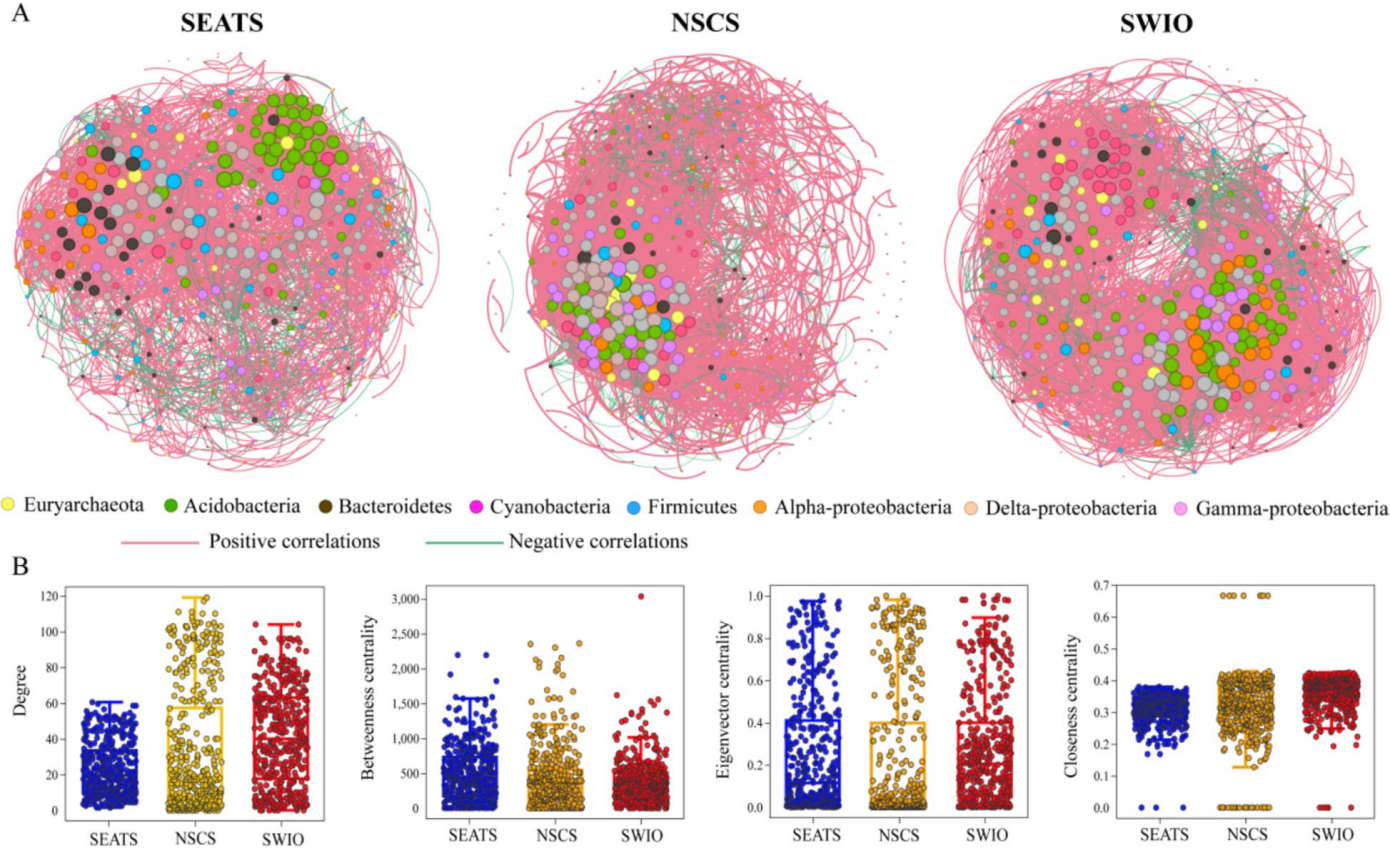
Distinct patterns of the prokaryotic co-occurrence networks in the three sampling regions. **A** Co-occurrence network analysis of the three regions. The size of the nodes corresponds to their rank order of degree. The nodes are connected by pink or green edges, which indicate significantly positive or negative relationships (estimated by Spearman’s correlation with a significance level of 0.01), respectively. The absolute values of the correlation coefficients represent the correlation strength, such that thicker edges indicate stronger correlations. **B** Network topological parameters for these habitats. NSCS indicates Haima and Site F, whereas SWIO indicates Longqi

A network co-occurrence analysis revealed the dominance of positive correlations in the three regions, with modularity indices of 0.63, 0.37, and 0.57 in the SEATS, NSCS, and SWIO, respectively (Fig. 4A). Higher modularity suggests a higher complexity of prokaryotic communities. The highest average degree of networks occurred in the SWIO communities, exhibiting the highest level of connectivity between the microbial taxa. Additionally, there was a much higher average clustering coefficient and path length in the NSCS (Fig. 4B) than in either SEATS or SWIO, and the microbial communities were more tightly grouped into clusters with more complex interactions between different taxa. Pearson correlation coefficient analysis demonstrated that significantly negative and positive correlations were found between the POC content and energy metabolism and between xenobiotics biodegradation and metabolism, respectively (Fig. S3A). For taxonomic compositions, Cyanobacteria and Firmicutes were significantly positively correlated with POC content (*p* < 0.01) (Fig. S3B). In addition, the Mantel test showed that POC was strongly correlated both with taxonomic (*r* > 0.7, *p* < 0.05) and functional (*r* > 0.7, *p* < 0.01) composition (Fig. S3C).

### Major metabolic processes of the microbiomes

**Fig. 5:**
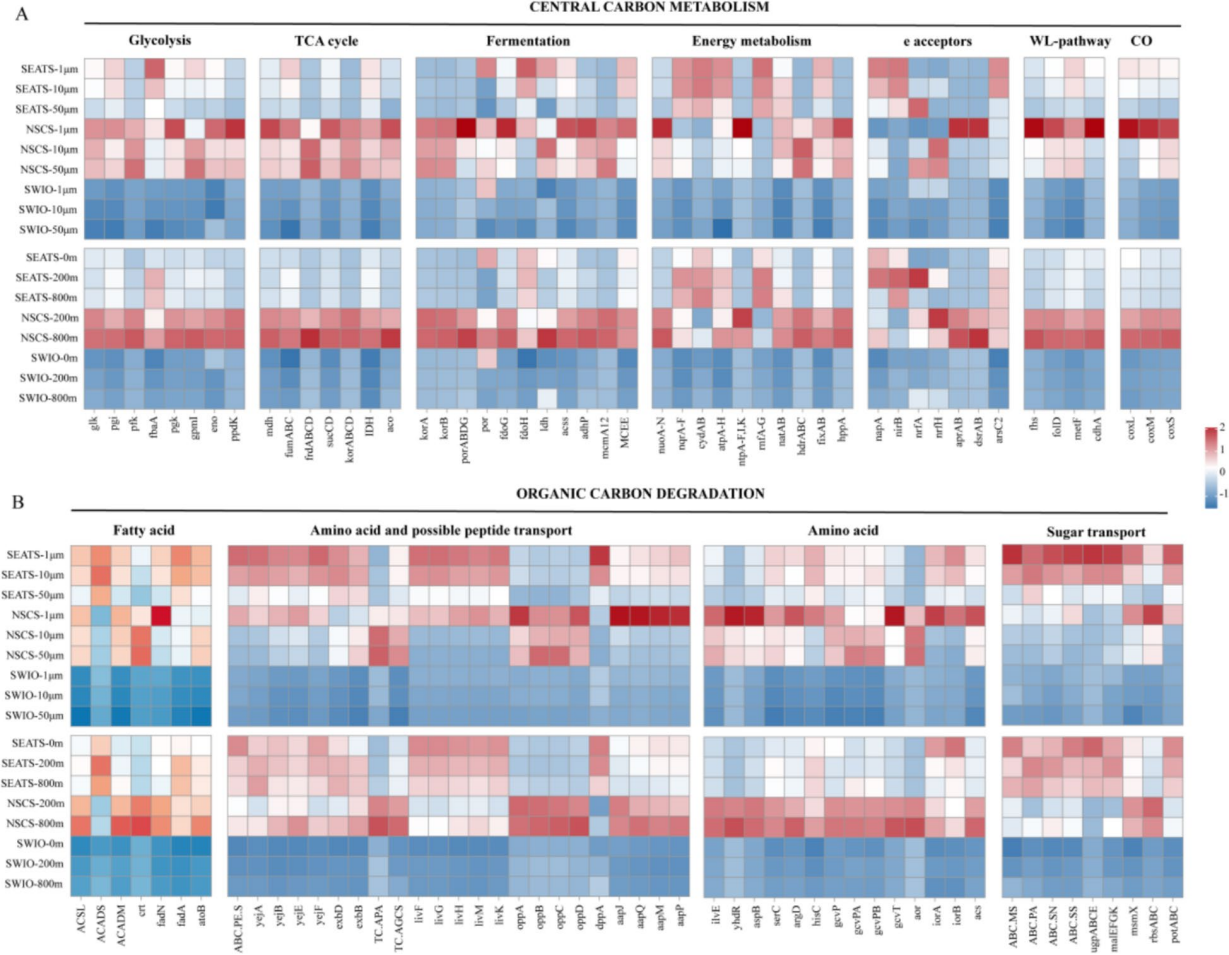
Z-score heatmap boxes showing **A** prokaryotic central carbon metabolic process genes and **B** organic carbon degradation-related genes in the different size fractions and water depths of the three regions. In **A**, “WL pathway” indicates genes of the Wood-Ljungdahl pathway of acetogenesis, and “CO” indicates carbon monoxide processing genes. NSCS represents Haima and Site F, whereas SWIO represents Longqi. The source data are listed in Table S7

The genes involved in organic carbon degradation and carbon metabolic processes were explored in further detail. A higher gene abundance related to carbon metabolic processes, such as glycolysis, the TCA cycle, the Wood-Ljungdahl pathway of acetogenesis, and the carbon monoxide process, was generally found in the NSCS, especially at the lower boundary and in the 1–10-μm fraction of the twilight zone (Fig. 5A). In contrast, a significantly lower gene abundance for organic carbon degradation was detected in the SWIO. Genes related to “amino acid and possible peptide transport” as well as “amino acid” and “sugar transport” were more frequently found in the 1–10-μm fraction (Fig. 5B).

**Fig. 6:**
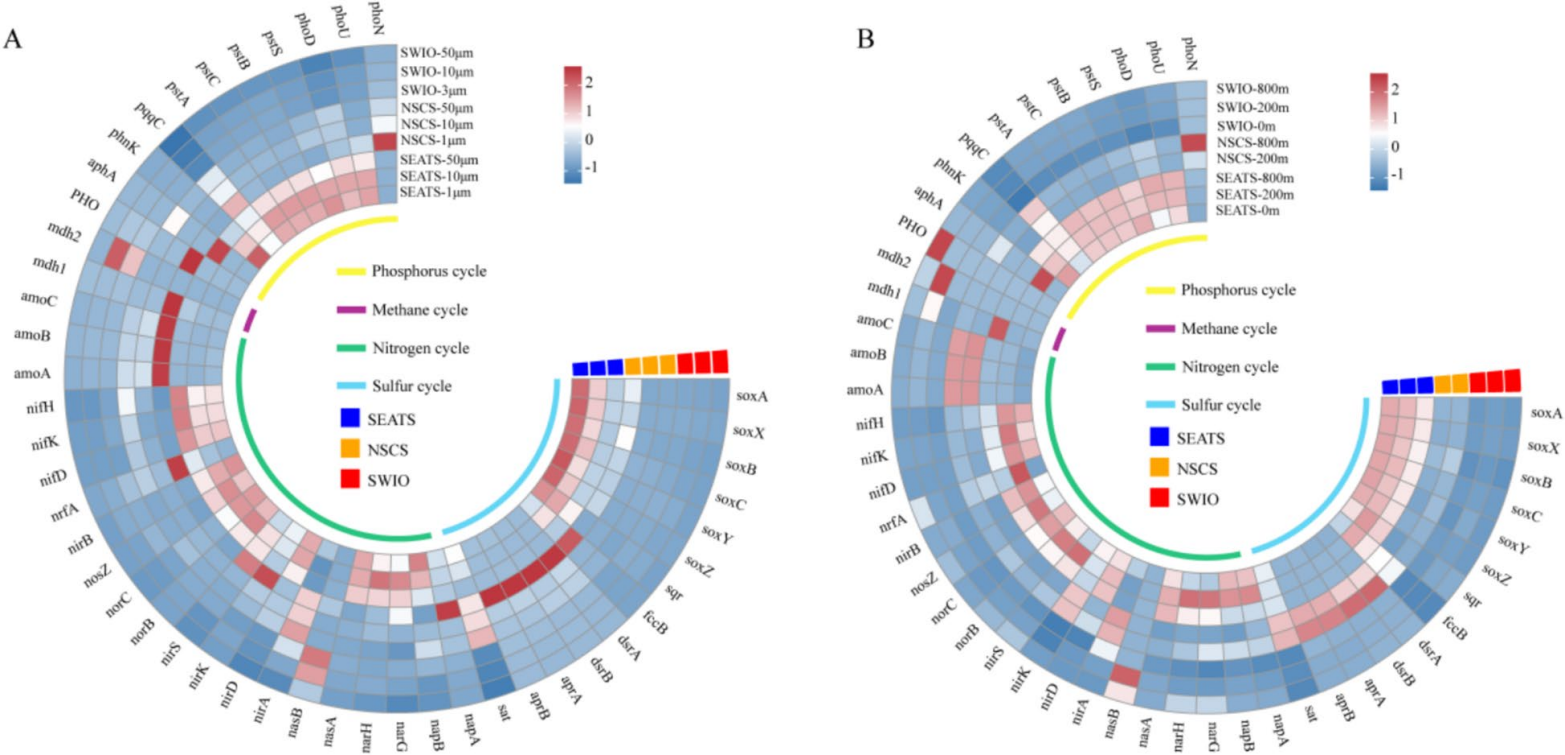
Z-score heatmap boxes showing the functional genes involved in the phosphorus, methane, sulfur, and nitrogen cycles (based on the KEGG database) in the different **A** size fractions and **B** water depths of the three regions. NSCS represents Haima and Site F, whereas SWIO represents Longqi

The essential metabolic processes in the different-sized fractions (Fig. 6A) and water depths (Fig. 6B) were also identified. A relatively high abundance of key nitrogen fixation genes (i.e., *nifH/D/K*) was detected in > 50-μm particles in shallow seawater (i.e., in the surface waters and at the upper boundary of the twilight zone), especially in the NSCS (Fig. 6A). Moreover, the abundance of ammonia-oxidizing genes (i.e., *amoA/B/C*) increased significantly with depth (Fig. 6B), and it was the highest in the NSCS at 800 m, especially in the 1–10-μm particles (Fig. 6A). In addition, *nxrA/B* (a key gene for nitrite oxidation) and both *norB/C* and *nosZ* (key denitrification genes) were highly abundant in the SEATS, whereas *norB/C* and *nosZ* had the lowest abundance at the lower boundary of the SWIO (Fig. 6B). Furthermore, *dsrAB*, *aprAB*, and *sat* (heterogeneous sulfate REDOX key genes) and *sox* (a SOX oxidation system gene) were more abundant in both NSCS and SEATS and were mainly concentrated in the 1–10-μm particles.

A series of nMDS analyses were performed based on carbon (Fig. S4A), methane, sulfur, and nitrogen (Fig. S4B) metabolic process genes. We found that in the NSCS, the function of the prokaryotic community in the 1–10-μm fraction was distinct from that of the other larger particles (Fig. S4A). Moreover, regarding methane, sulfur, and nitrogen function, the SWIO exhibited a distinct functional composition among the different water depths (i.e., the surface, upper and lower boundary layers of twilight zone) (Fig. S4B).

### Recovery of MAGs and phylogenetic and functional annotation

**Fig. 7:**
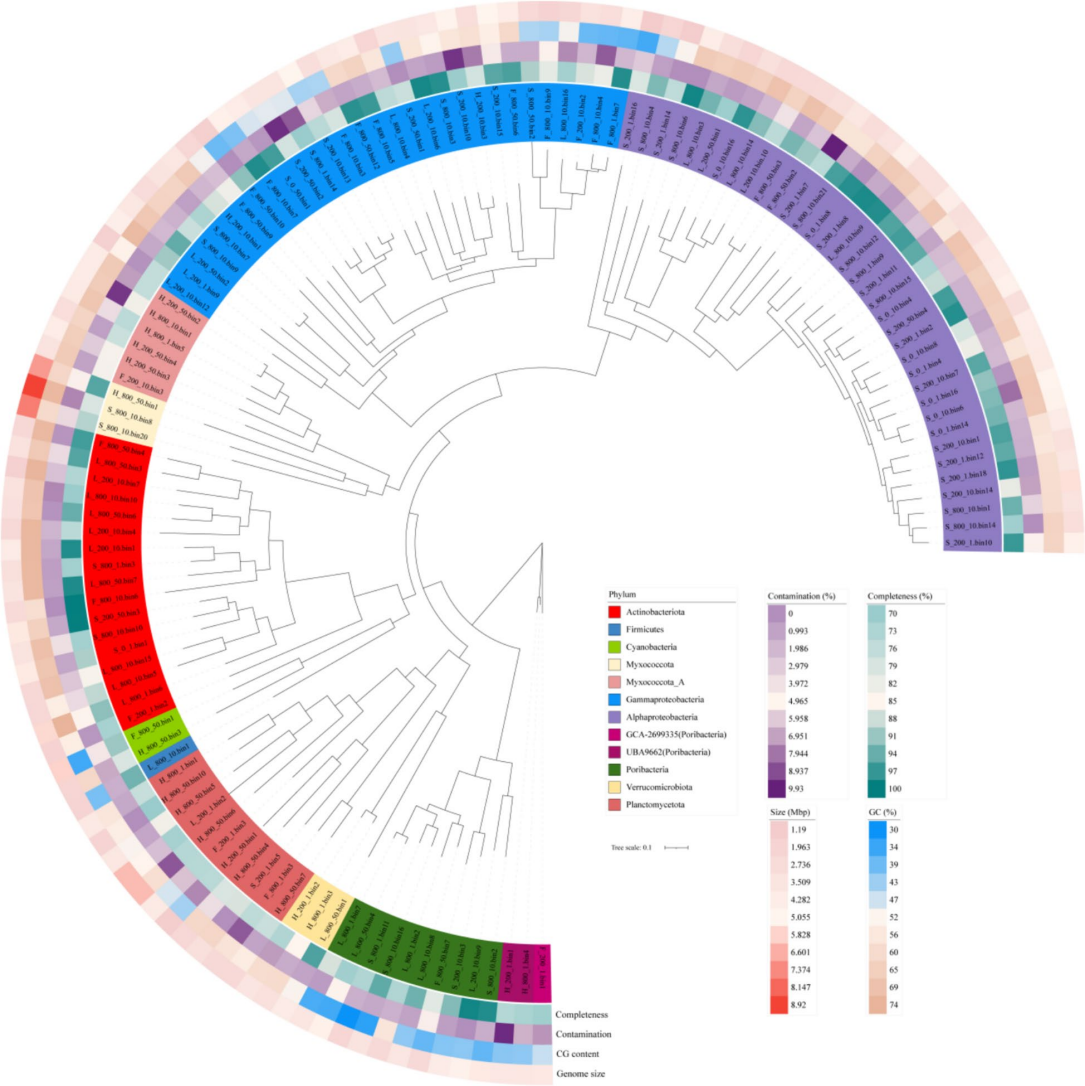
Phylogenetic tree constructed from the 122 retrieved MAGs. The outer layers of the tree indicate (from inside to outside) the color codes of the various bacterial phyla, as well as the percentage completeness, contamination, GC content, and genome size of each MAG

**Fig. 8:**
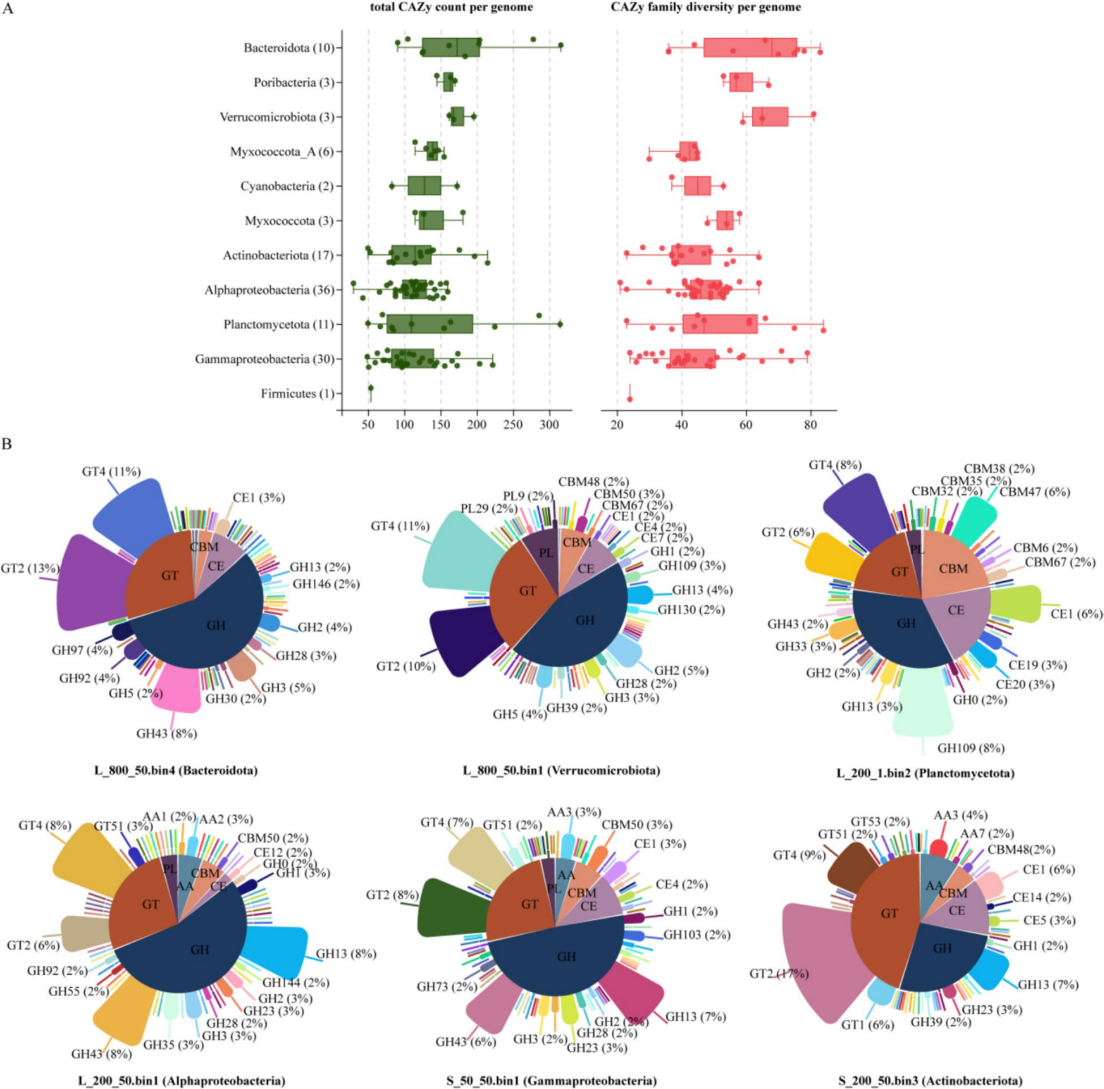
Composition and diversity of the carbohydrate-active enzymes (CAZyme) in each MAG across the microbial taxa. **A** Boxplots showing the total CAZy gene count per MAG within each phylum (green) and the CAZyme functional diversity (i.e., number of CAZy families per MAG; red). The boxplots indicate the median values as well as the lower and upper quartiles, and the numbers in brackets on the *y*-axis denote the MAG count. All the CAZymes belonging to the AA, CBM, PL, CE, GH, and GT classes were considered. **B** Distribution of CAZymes across the MAGs of six major bacterial phyla. AA, auxiliary activities; CBM, cellulose-binding motifs; CE, carbohydrate esterases; GH, glycosyl hydrolases; GT, glycosyltransferase; and PL, polysaccharide lyases

After removing redundancies, 122 dereplicated high-quality MAGs were obtained. Proteobacteria (*n* = 66), Actinobacteriota (*n* = 17), Bacteroidota (*n* = 10), and Planctomycetota (*n* = 11) were the major bacterial phyla found (Fig. 7, Table S3). The highest values of GC content and the largest genome size occurred in L_800_1.bin6 (*Sphingopyxis*, 74.08%) and S_800_10.bin8 (*Roseibacillus*, 8.92 Mbp), respectively (Fig. 7). The ANI values of pairwise comparisons and the relative abundance of the dereplicated MAGs were described in Tables S4 and S5, respectively. Protein coding annotations based on the KEGG database revealed that carbohydrates, amino acid, energy metabolism, and the metabolism of cofactors and vitamins were ubiquitous in the MAGs (Table S6). The highest numbers of total CAZy count per genome and CAZy family diversity per genome were both found in Bacteroidota (Fig. 8A). A series of CAZymes across the MAGs from six major bacterial phyla were identified, especially for glycosyltransferase (GT) involved in glycan synthesis and modification and glycosyl hydrolases (GH) as diverse carbohydrate-active enzymes that target *α*- and *β*-linked polysaccharides (Fig. 8B).

## Discussion

### Effect of particle size on the microbial community

Our comparative study found that particle size rather than sampling depth had a more profound influence on the particle-associated prokaryotic community in the SCS. Our findings agree with previous observations made in the waters off the coast of Iquique, Chile [20]. For example, we found that the photoautotrophic Cyanobacteria were more enriched in the larger-sized particles collected from the SCS (Fig. 2). Similarly, in the Eastern Tropical South Pacific off the coasts of Chile and Peru, which is a marine oxygen minimum zone, Cyanobacteria were also more abundant in the large size fractions [29]. On the other hand, we found that Alphaproteobacteria preferred the smaller particles (Fig. 2). This group has previously been identified as being particle-preferring bacteria [30], which are enriched in small particles [31]. We also showed that microbes associated with the smallest (1–10 μm) particles possessed a lower diversity (Fig. 2) along with higher metabolic potentials for carbon (Fig. 5), phosphorus, methane, sulfur, and nitrogen (Fig. 6). This might be because the larger particles are likely to consist of multiple compartments with a more complex distribution of resources, to meet the requirement of a broader range of microbial communities.

Regarding the potential metabolic functions of different-sized particles, the microbes that attached to the smallest particles exhibited a higher abundance of the branched-chain amino acid ABC transporter (*livGHMK*), sugar transport (e.g., ABC.PA, ABC.SN, and ABC.SS), and formate dehydrogenase (*fdoG*) genes than those attached to the larger particles (Fig. 5). The microbial communities associated with these small particles also contained a full pathway for fatty acid degradation and the utilization of propanoate and butanoate (Fig. 5). This indicates the increased likelihood of the smaller particles being degraded by their associated microbiota when they pass through the twilight zone [32]. This is because the organic matter contained in smaller particles is less likely to be transported to the bottom of the ocean for long-term sequestration and more likely to be recycled. Indeed, it was recently reported that small, suspended particulate organic matter is also the “primary site” for microbial activity in the twilight zone of the Scotia Sea [33]. Moreover, in a previous biogeochemical study, particles of 1–10 μm were shown to contain the largest proportion of POC (26–35%) within the trap-collected POC pool in the NSCS [27], once again indicating their importance in metabolizing POM in the twilight zone.

### Niche differentiation of the microbial community

Distinct prokaryotic communities with significantly lower diversity and endemic species were detected in the SEATS compared to the other two regions (Fig. 2). This significant difference in biogeographic distribution might be caused by variations in the *in situ* physicochemical conditions and availability of labile organic matter [34]. Mesopelagic ecosystems depend on the input of detritus from primary producers in the upper layers [35]. The production of organic matter through phytoplankton photosynthesis in the euphotic zone, and its subsequent export to the deep layers through various pathways, is the main source of food and energy for communities in the twilight zone [36, 37]. For this reason, the quantity and quality of the organic matter delivered from the upper ocean can directly regulate the size, structure, and function of the microbial communities in this zone [38]. We suggest that the relatively low levels of nitrate, phosphate, and POC detected in the SEATS surface water might help to explain the distinctive microbial community structures in both the surface water and the twilight zone of this region when compared with the other regions.

The microbial community structure and diversity of the surface layer were found to be significantly different from those of the twilight zone, especially in the SWIO (Fig. 2). This might be because phototrophs only inhabit the surface sunlit layer, whereas the microbiota in the twilight zone mainly participate in the degradation, transformation, and remineralization of POC [39]. Our cluster analysis showed that the particle-attached microbial communities sampled in the surface water were significantly different from those collected at 200 m. This suggests that the microbial community in the twilight zone is largely determined by the prokaryotic colonization of particles, rather than those “inherited” from the euphotic zone.

The vertical difference in microbial community structure between the upper and lower boundaries of the twilight zone might be related to the size and chemical characteristics of the sinking material [40]. Particles have a highly heterogeneous organic and inorganic composition, and they undergo changes as they sink [12, 41]. It is the composition of particles that determines the colonization and structure of the initial microbial communities [42]. In our current study, a decreasing proportion of Bacteroidetes in the twilight zone was found. This might be linked to the characteristic decrease in POM with water depth. Bacteroidetes are frequently found on particles of organic matter, and they are known to specialize in the degradation of high-molecular-weight compounds in the particulate fraction of the marine organic matter pool [43, 44].

Microbes such as Alteromonadales, which secrete enzymes into the environment, were dominant at the lower boundary of the twilight zone, where an increase in refractory compounds results from the microbial utilization of labile organic matter as particles descend [45]. Among all the taxa that sink with particles, the ability of Alteromonadales to thrive in the deeper layers might depend on the origin of the particles in the surface waters [46]. Indeed, here we observed significant community shifts on sinking particles and vertical connectivity between the euphotic zone and the twilight zone. Although small primary producers such as *Prochlorococcus* and *Synechococcus* were dominant in both NSCS and SWIO, they were found with significantly greater abundance in the surface water of the latter. The low enrichment of attached Cyanobacteria in the twilight zone of both NSCS and SWIO suggests that most of these small cells were either consumed by micrograzers in the surface water or else they could not sink effectively.

The higher abundance of carbon degradation-related genes found at the lower boundary of the twilight zone than at the upper boundary indicates that prokaryotes in the former might be stimulated by abundant substrates [47]. This finding suggests that carbon degradation becomes increasingly important in shaping the microbial community structure in the deeper waters. The abundance of carbon degradation-related genes at the lower boundary might also result from recalcitrant organic components in the POM, which accumulate when they sink, given that biodegradable components are continuously consumed by the surrounding bacteria [48]. We also found a higher relative proportion of recalcitrant carbon degradation-related genes at the lower boundary of the twilight zone (Fig. S5), which suggests that particle-associated microbial communities with versatile hydrolytic enzymes might become increasingly important in this location. Continuous degradation by particle-attached prokaryotes alters the chemical and biological properties of the POM [49], which consequently affects the microbial community structure with associated function [50, 51]. In our study, the clear shift in microbial community composition and metabolic functions between the upper and lower boundaries of the twilight zone suggests that the colonization and mineralization of microbes during the POM sinking process might promote the transformation of POM, which in turn alters the microbial community structure and/or associated function.

### Ecological function of the microbes in the twilight zone

The SEATS station is located in the basin of the SCS. In this region, heterotrophic prokaryotes might benefit from producing exopolymeric material to facilitate their scavenging of organic matter from the environment [52]. As predicted, this region was dominated by the Alteromonadales, Rhodobacterales, and Oceanospirillales; these are the main degraders of transparent exopolymer particles and chitins, both of which are major constituents of POC [45]. These microbes produce exopolysaccharides to trap nutrients and retain extracellular enzymes for the degradation and transformation of organic material, as their associated particles sink through the water column [53]. In contrast, in the NSCS and SWIO, more diverse prokaryotic groups were found in particles of all size ranges when compared with those in the SEATS. These include Planctomycetales, which degrade sulfated polymeric carbon [54] (e.g., marine snow); the heterotrophic Verrucomicrobiales, which degrade carbohydrates [55]; and Sphingomonadales, which are generally associated with marine snow and phytoplankton blooms [56].

The MAGs recovered in our study showed diverse taxonomic and metabolic strategies throughout the twilight zone and exhibited complex relationships in mediating carbon, nitrogen, and sulfur cycling processes (Table S6). In the euphotic zone, photosynthetic Cyanobacteria produce organic carbon via photosynthesis; in the twilight zone, the chemoautotrophic *Rubrivivax* (Grammaproteobacteria) and *Marinovum algicola* are involved in potential ammonia and sulfur oxidization metabolisms. Moreover, along the water column, the process of remineralizing organic particles to form inorganic compounds was facilitated by diverse microorganisms, such as Acidobacteriota, Bacteroidota, and Alpha/Grammaproteobacteria.

Members of the Bacteroidota are potential key players in carbohydrate metabolism as we found that they have the highest total CAZy count and family diversity among all the constituents of the particle-associated microbial communities. In addition, they appeared to be more capable of utilizing complex and diverse carbohydrates than the other taxa. This is in line with the known ability of many Bacteroidota to degrade polysaccharides in marine environments and mammalian gut systems [57]. Consistent with our contig-based results, abundant genes that encode carbohydrate-metabolizing enzymes, which drive organic carbon degradation, were contained in the MAGs. GHs (mainly GH43 and GH3) and GTs (mainly GT2 and GT4) were more abundant in the Bacteroidota MAGs and likely catalyze the degradation of various complex carbohydrates, such as cellulose, mannan, xylan, chitin, and starch [58], and the synthesis of glycosidic bonds by harnessing activated sugar donors to many acceptor substrates [59]. Poorly understood members of Verrucomicrobiota and Poribacteria also demonstrated a high potential capacity for extracellular polysaccharide degradation in the twilight zone. Together, our metagenomic analysis extends the understanding of microbial carbon degradation pathways in the twilight zone, with Bacteroidota, Verrucomicrobiota, and Poribacteria being highlighted as potentially important players.

## Conclusions

We conducted a comprehensive analysis of the microbial communities associated with sinking particles of different sizes, focusing on the transitions in their ecological functions across the twilight zone. Our results indicated that microbial communities play a crucial role in the remineralization of POC. Notably, microbial assemblages of the surface and twilight zone in the open ocean (SEATS) were markedly distinct from those of the other sampling regions (i.e., the NSCS and the SWIO). This difference can likely be attributed to *in situ* physicochemical conditions and the characteristics of the particles, which in turn can be linked to the trophic conditions of the surface water. Our study also revealed significant shifts in microbial community composition and function in the twilight zone, with a clear particle size effect observed. Microbes exhibit different responses to the POC entering the twilight zone, and they potentially drive the transformation of POC through this zone. We found that the smaller particles play a more critical role in the degradation of organic matter than the larger ones. This might be due to the smaller particles being able to facilitate greater microbial colonization and utilization, thereby enhancing the efficiency of organic matter degradation and carbohydrate metabolism.

In this study, we attempt to entangle the key role of microbes in the remineralization of POC in the twilight zone, in addition to the well-known important role of zooplankton. We admit that solely relying on genomic DNA analysis could not provide the actual metabolic clue of the microbiomes. Future studies acquiring more in-depth information from metatranscriptomics and incorporating isotopic tracing and incubation experiments will provide an overall comprehensive understanding of the active metabolic pathways, gene expression profiles, and biogeochemical processes of microbial communities occurring within the twilight zone.

## Materials and methods

### Sample collection, genomic DNA extraction, and sequencing

Water samples were collected from the surface (i.e., 0 m) as well as the upper and lower boundaries of the twilight zone (i.e., ca. 200 and 800 m) in the South East Asian Time-series Study station (14°00′N, 116°30′E, South China Sea (SCS), SEATS) and above two cold seep sites (i.e., Haima, 16°43′N, 110°28′E and Site F, 22°6′N, 119°17′E, northern South China Sea (NSCS)) in 2018, as well as above a hydrothermal vent (Longqi, 37°46″S, 49°38′E, Southwest Indian Ocean (SWIO)) in 2019 (Fig. 1). Approximately, 500-L seawater were collected for each sample and filtered sequentially through polyester meshes of pore size 50 µm and 10 µm, followed by a quartz microfiber filter (QMA) of pore size 1 µm, using a Multiple Unit Large Volume *in situ* Filtration System (MULVFS) [60]. Correspondingly, three size fractions (i.e., > 50 µm, 10–50 µm, 1–10 µm) of particle-attached microbial community data were collected. All the filters were immediately flash frozen and stored at − 80°C until further analysis.

The genomic DNAs were extracted from the various filter membranes with the PureLink Genomic DNA kit (Invitrogen, Carlsbad, CA, USA), following the manufacturer’s instructions. The concentration of genomic DNA acquired was quantified using a Qubit 2.0 Fluorometer (Invitrogen, Life Technologies), and the quality was checked via gel electrophoresis. The DNA extracted in a total volume of 50 µL in a 0.5-mL microcentrifuge tube was fragmented to 350 bp using Covaris Adaptive Focused Acoustics. The fragments were then end-polished and A-tailed following standard Illumina protocols, after which they were ligated with adaptors for Illumina sequencing with further PCR amplification and purification using the NEBNext Ultra II kit (New England Biolabs). After construction of the library, an Agilent 2100 Bioanalyzer system (Agilent Technologies, Santa Clara, CA, USA) was used to detect the inserted size of the library. Sequencing was performed with an Illumina NovaSeq 6000 PE150 platform (Novogene Co., Ltd., www.novogene.com).

### Chemical analysis of the seawater samples

*In situ* hydrographical parameters (i.e., location, temperature, depth, and salinity) were recorded at each station with a conductivity-temperature-depth (CTD) rosette system (Sea-Bird Electronics). In addition, the concentration of inorganic nutrients (i.e., nitrate, silicate, ammonia, and phosphate) was analyzed with an auto-analyzer (QuAAtro, BLTEC. Co. Ltd.), which was calibrated with certified seawater nutrient reference material (RM; KANSO). Approximately, 8-L seawater was filtered through Whatman glass fiber filters (GFF), which had been pre-combusted in a muffle furnace at 500°C for 12 h. After filtration, the filters and filtrates (volume of 30 mL) were both stored at − 80°C for POC and dissolved organic carbon (DOC) analysis, respectively. The DOC and POC concentrations were measured using an organic carbon analyzer (TOC-L CSH CSN; Shimadzu Corp., Japan) with an accuracy of 0.01 mM [61] and an elemental analyzer (Vario EL Cube, Elementar, Germany) [62], respectively.

### Quality control and metagenome assembly

The sequencing data were first assigned to each sample according to its barcodes. After being trimmed to remove the adapter, the quality of the sequences was controlled to provide a length > 140 bp, without ambiguous base “N,” and an average base quality > 20 using the FASTX-Toolkit [63], and it was subsequently checked by FastQC (Babraham Bioinformatics). All the taxonomic and functional annotations were based exclusively on the quality reads. High-quality short reads from each sample were de novo assembled using MEGAHIT version 1.2.9 [64] with kmer 55, -d 1, -M 3, -R, -u, and -F.

### Prokaryotic taxonomic assignment and functional annotation

To further confirm the composition of the microbial community, rRNA sequences of the small subunit (SSU) were extracted from the Illumina data using Metaxa2 version 2.2.2 [65]. The SSU rRNA reads obtained were then analyzed with QIIME 2 [66]. Taxonomy assignment of the SSU rRNA reads was determined against the SILVA 138 database [67], following the analysis pipeline described by Caporaso et al. [66]. An analysis of similarities (ANOSIM) was conducted with Paleontological Statistics (PAST) version 3.19 [68], to test if there were any significant differences in the microbial communities among the various sampling regions.

The open reading frames (ORFs) of the assembled contigs were identified by MetaGeneMark version 3.38 [69] with default parameters and then clustered using CD-HIT version 4.6.8 [70] with -c 0.95, -G 0, -aS 0.9, -g 1, and -d 0, to remove sequence redundancy and improve the performance of sequence analyses. The clean reads were mapped back to the predicted genes using Bowtie 2.2.9 [71] with -end-to-end, -sensitive, -I 200, and -X 400, to obtain an accurate value for the abundance of each gene. Functional annotations of the assembled scaffolds (unigenes) were performed using the Kyoto Encyclopedia of Genes and Genomes (KEGG) release 90.1 database [72] and the nonredundant protein (NR) database, via the Diamond software [73] using BLASTP with *E*-value < 10^−10^ parameters. Searches for CAZymes [74] were performed as described by Liu et al. [75], to identify genes encoding carbohydrate metabolizing enzymes.

### Genome binning and annotation

Genome binning was performed using CONCOCT (v1.0.0), MaxBin (v2.2.6), and MetaBAT (v2.12.1) via the MetaWRAP pipeline (v1.3.2) [76]. All the MAG sets were pooled together and dereplicated using dRep v2.3.2 [77]. In addition, CheckM (v1.1.3) [78] was used to assess the completeness and contamination of the retrieved MAGs. We obtained 122 MAGs that met the threshold of medium quality (i.e., > 70% completeness and < 10% contamination). The average nucleotide identity (ANI) values of pairwise comparisons of these genomes were evaluated by FastANI [79]. The relative abundance of the dereplicated MAGs was calculated using CoverM (v 0.7.0) (https://github.com/wwood/CoverM), which mapped metagenomic sequences to the MAGs with default parameters. Their taxonomy was assigned using GTDB-TK (v2.0.0) [80] with GTDB release 207 using default parameters. Finally, functional annotations of the MAGs were performed using the KEGG 90.1 database [72] and the HMM database V9 of dbCAN2 [81].

### Statistical analysis

The UPGMA and nMDS based on the Bray–Curtis similarity index were used to study the distribution pattern of the microbial communities within PRIMER 5 (Plymouth Marine Laboratory, West Hoe, Plymouth, UK) [82]. The relative microbial abundance was analyzed using the Kruskal–Wallis test with false discovery rate (FDR) correction for multiple testing. The key bacterial order responsible for discrimination between the different groups was identified using the linear discriminant analysis (LDA) effect size (LEfSe). *LDA* > 3.5 and *P* < 0.01 indicated significantly enriched microbial communities [83]. According to the relative abundance of prokaryotes at the order level, significant differences between the upper and lower boundaries of the twilight zone were calculated by the two-sided Welch’s *t*-test with Benjamin-Hochberg false discovery rate (FDR) correction, using the Statistical Analysis of Metagenomic Profiles (STAMP, version 2.1.3) [84], and the results were displayed by extended error bar plots using the “ggplot2” packages in R (version 4.3.3).

Network analysis was conducted to explore the co-occurrence patterns within/between different taxonomic groups. Utilizing a similarity matrix generated by a taxonomy table, the correlation matrix, *r*- and *p*-values were calculated using the “psych” R package [85]. Statistically significant correlations (Spearman’s |r|> 0.8 and FDR-adjusted *p* < 0.01) were further visualized with Gephi version 0.9.3 [86]. Various network properties, such as the average degree, path distance and clustering coefficient, and the modularity index, were characterized based on similarity matrices. The Mantel test was performed to compare the responses of taxonomic and functional compositions to environmental variables.

## Supplementary Information

Supplementary Material 1.

## Data Availability

No datasets were generated or analysed during the current study.
